# Multifunctional Hydroxyapatite Coated with *Arthemisia absinthium* Composites

**DOI:** 10.3390/molecules25020413

**Published:** 2020-01-19

**Authors:** Mariana Stefania Raita, Simona Liliana Iconaru, Andreea Groza, Carmen Cimpeanu, Gabriel Predoi, Liliana Ghegoiu, Monica Luminita Badea, Mariana Carmen Chifiriuc, Luminita Marutescu, Roxana Trusca, Ciprian Florin Furnaris, Claudiu Stefan Turculet, Dorin Valter Enache, Daniela Predoi

**Affiliations:** 1 Faculty of Veterinary Medicine, University of Agronomic Sciences and Veterinary Medicine of Bucharest, 105 Splaiul Independentei, Sector 5, 050097 Bucharest, Romania; raitastefania@gmail.com (M.S.R.); gabrielpredoi2017@gmail.com (G.P.); ffurnaris@gmail.com (C.F.F.); 2 Multifunctional Materials and Structures Laboratory, National Institute of Materials Physics, Atomistilor Street, No. 405A, P.O. Box MG 07, 077125 Magurele, Romania; simonaiconaru@gmail.com (S.L.I.); lilianaghegoiu73@gmail.com (L.G.); monibadea78@gmail.com (M.L.B.); 3 Low Temperature Plasma Laboratory, National Institute for Laser, Plasma and Radiation Physics, 409 Atomistilor Street, P.O. Box MG 36, 077125 Magurele, Romania; andreeagroza75@gmail.com; 4 Faculty of Land Reclamation and Environmental Engineering, University of Agronomic Sciences and Veterinary Medicine of Bucharest, 59 Marasti Blvd, Sector 1, 011464 Bucharest, Romania; carmencimpeanu@yahoo.com; 5 Faculty of Horticulture, University of Agronomic Sciences and Veterinary Medicine, 59 Marasti Blvd., 011464 Bucharest, Romania; 6 Microbiology Department, Faculty of Biology, University of Bucharest, 1–3 Portocalelor Lane, 77206 Bucharest, Romania; carmen.chifiriuc@gmail.com (M.C.C.); lumi.marutescu@gmail.com (L.M.); 7 Earth, Environmental and Life Sciences Section, Research Institute of the University of Bucharest (ICUB), 91-95 Splaiul Independentei, 050095 Bucharest, Romania; 8 National Centre for Micro and Nanomaterials, University Politehnica of Bucharest, 060042 Bucharest, Romania; truscaroxana@yahoo.com; 9 Carol Davila University of Medicine and Pharmacy, 8 Eroii Sanitari, Sector 5, 050474 Bucharest, Romania; c.s.turculet@gmail.com; 10 Faculty of Food and Tourism, Transilvania University of Brasov, 29 Eroilor Blvd., 500036 Brașov, Romania; doenache@yahoo.com

**Keywords:** hydroxyapatite, *Arthemisia absinthium*, antimicrobial properties

## Abstract

There is significant research showing that essential oils extracted from the plants have antibacterial effects. The purpose of this study was to develop a biocomposite based on hydroxyapatite coated with *Artemisia absinthium* essential oil and to highlight its antibacterial activity. Therefore, present studies are aimed at developing new materials combining hydroxyapatite with *Artemisia absinthium* essential oil, in order to avoid postoperative infections. The purpose of this work is to highlight the antimicrobial properties of the *Artemisia absinthium* essential oil-hydroxyapatite composites obtained by a simple method and at low costs. The structural properties and antimicrobial efficiency of the *Artemisia absinthium* essential oil-hydroxyapatite composite have been studied. The samples based on *Artemisia absinthium* essential oil analyzed in this study showed that wormwood essential oil presented the highest efficacy against the fungal strain of *C. parapsilosis*. It has been shown that wormwood essential oil has a strong antimicrobial effect against the microbial strains tested in this study. Furthermore, the antimicrobial properties of the biocomposites based on hydroxyapatite and essential oil are due to the presence of the essential oil in the samples.

## 1. Introduction

In recent years, the alarming increase of drug resistant bacterial strains have directed researchers to give special attention to the development of unconventional alternatives for treating bacterial infections [1,2]. The most pronounced bacterial infections are those related with hospitals equipment and improper wound dressings. The most studied species of Gram-positive and Gram-negative bacteria deemed responsible for severe infections are being considered *Staphylococcus aureus* and *Escherichia coli* strains. *S. aureus* is known to produce numerous diseases like skin infections, food poisoning, as well as endocarditis and bloodstream infections [3,4]. According to the literature, *E. coli* causes severe urinary tract infections and enterocolitis [3,4]. Recently, due to the progress made in the biomedical field the conventional natural and synthetic wound dressings are being replaced by new and improved dressings made of textiles, gels, and various materials that possess biocompatible properties and are able to deliver active substances locally to facilitate wound healing [5]. A survey conducted by the World Health Organization (WHO) showed that 2/3 of the world’s population still rely on traditional healing, using plants and herbs to treat various diseases. During the last decades, modern medicine has taken a serious stance on studying plants and their extracts trying to explain and demonstrate their healing properties in a scientific manner. Even though herbs have been used since the beginning of time for their medicinal properties, human knowledge has barely scratched the surface on how plants influence our physiology and health and much research must be done in order to fully understand their real potential [6]. Essential oils (EOs) extracted from different medicinal plants have been used since ancient times as natural remedies to combat numerous pathogens [7]. *Artemisia absinthium* L. belonging to the *Asteraceae* family, commonly known as “wormwood” in United Kingdom, as “absinthe” in France, and as “chajret mariem” in Tunisia has been known since ancient times as having important botanical and pharmaceutical properties [8,9]. During the years, wormwood essential oil has been widely used in traditional medicine due to its antimicrobial [10], insecticidal [6], neuroprotective [11], acaricidal, antimalarial [11], hepatoprotective [12], and antidepressant [13] properties. In addition, the organic extract of this herb has been reported to exhibit a toxic and antifeedant effect against *Leptinotarsa decemlineata* [14].

Wormwood essential oil exhibited strong anthelmintic action. In addition to its well-known antiparasitic activity, wormwood extracts also showed antioxidant and phytotoxic effects [6]. Wormwood is effective against both bacteria and fungi, signaling a good antimicrobial activity—its essential oil inhibited the development of wide-ranging microorganisms [9]. Wormwood exhibits adaptogenic and nootropic activity, thus enhancing memory, improving thinking and alertness in patients suffering from conditions affecting the mind. In an experiment conducted on rats with induced diabetes, it was observed that wormwood extracts displayed remarkable antidiabetic properties [9,10,11,12,13,14,15,16,17]. It also reduced the elevated triglycerides, creatinine levels and total cholesterol while increasing the HDL cholesterol. Another study noted that wormwood presented antiproliferative effects on human breast cancer cells by inducing apoptosis on affected cells. Moreover, displayed remarkable gastrointestinal effects by restoring stomachal juices and normalizing the digestive functions, stimulating the appetite, aiding the lipid digestion and the absorption of nutrients thus promoting gut health [13,17,18]. It also partially inhibited the induced hyperperistalsis in rats. Being a rich source of flavonoids, terpenes and other compounds, wormwood extracts have been studied for their anthelmintic and antioxidant properties and modern medicine noted that its biologically active compounds can neutralize the free radicals and toxins and kill gastrointestinal parasites [18]. Wormwood essential oil contains a compound called thujone which stimulates the central nervous system and acts as a tonic for nerves. It was found that it also improves blood circulation [10]. The decoction of wormwood leaves is used in paralytic disorders, by improving the sensation and movement of paralyzed limbs. It is also used as an ayurvedic medicine for hysteria and epilepsy. In combination with other plant extracts such as *Melissa* and *Mori follium*, wormwood was demonstrated to suppress obesity. Moreover, wormwood has fertility regulator effects, by decreasing the negative results of stress on potency and fertility in mice [18].

Recent studies on *Artemisia absinthium* revealed the plant’s considerable antidepressant properties, mainly due to the high polyphenols content of its extracts [13]. Besides, wormwood oil and aqueous extracts hold worthy anti-inflammatory and antinociceptive properties—more like analgesic actions [19]. Being an excellent biocompatible and bioactive nanomaterial, hydroxyapatite (also known as HAp) is endorsed by biological studies as being a worthy carrier for low concentrations of essential oils, thus exhibiting enhanced antimicrobial actions [20].

Considering that hydroxyapatite has a very good biocompatibility and *Artemisia absinthium* has a high potential to act as an antimicrobial agent, in this study we aimed to make a biocomposite that can be used as a source for antimicrobial drugs. In our study, we examined the in vitro antibacterial activity of wormwood (*Arthemisia absinthium L.*) essential oil (WW EO), hydroxyapatite coated with wormwood essential oil (HApWW) and hydroxyapatite (HAp) against a series of Gram-negative bacteria (*E. coli* ATCC 25922, *E. coli* C5 (carbapenemase producer strain), *P. aeruginosa* ATCC 27853, *P. aeruginosa* ATCC 9027), Gram-positive bacteria (*S. aureus* ATCC 25923, *S. aureus* ATCC 6538 *E. faecium* DSM 13590), and a fungal strain *C. parapsilosis* ATCC 22019. The physico-chemical properties of the samples were also investigated.

## 2. Results and Discussions

The chemical composition of WW EOs obtained from dried root parts of *Arthemisia absinthium* L. was investigated by GC–MS. The results of the qualitatively and quantitatively GC-MS studies are presented in Table 1. The GC–MS analysis revealed 91.29% of the essential oils components and emphasized that the most abundantly chemical components found in the WW EO were α-Fenchene, Sabinene, β-Thujene, β-myrcene and methyl salicylate. Moreover, trace amounts of other chemical constituents have been identified.

Similar results regarding the elemental composition of essential oil extracted from roots of *Arthemisia absinthium* L. were reported by Kennedy et al. [21], who highlighted in their study that the major chemical constituent of the wormwood essential oil extracted from normal roots of *Arthemisia absinthium* L. was α-Fenchene. A considerable amount of α-Fenchene in the composition of WW EOs obtained from root parts of *Arthemisia absinthium* L. was also reported by Nin et al. [22] and by Blagojevica et al. [23]. The results of the GC-MS analysis obtained in this study suggested that the WW EO obtained from roots had as most abundant chemical constituents, monoterpenes.

The chemical composition of essential oils is usually variable and depends on numerous parameters such as geographical location, geology, climate, season of harvest, part of the plant and also the method used to obtain the essential oil.

**Figure 1:**
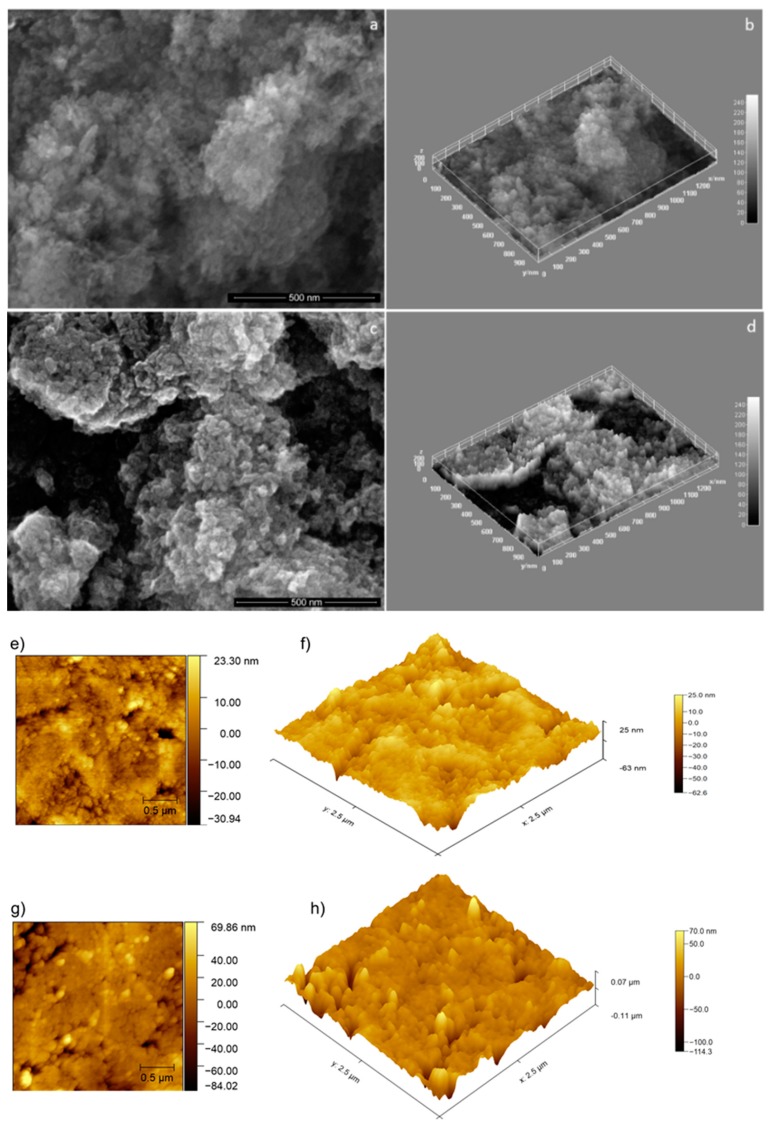
SEM images of HAp nanoparticles (**a**); 3D surface plot of SEM images of HAp (**b**); SEM images of HApWW sample (**c**); 3D surface plot of SEM images of HApWW sample (**d**) and AFM 2D and 3D surface topographies of HAp pellet (**e**,**f**) and HApWW pellet (**g**,**h**).

The influence of the adsorption of *Arthemisia absinthium* EO on the distribution and shape of HAp nanoparticles was analyzed by SEM. In Figure 1a,c can be observed the distribution of HAp nanoparticles into the powder before and after their immersion in *Artemisia absinthium* EO. By covering the particles with the EO, their shapes are unaffected. Only the particles agglomeration and their distribution into the powder are changed (Figure 1a,c).

The uniformity of particles distribution and their homogeneity in the investigated samples were analysed using Image J software (ImageJ 1.51j8, National Institutes of Health, Bethesda, MD, USA) [24]. Thus, in Figure 1b,d are showed the 3D surface plots of SEM images characteristic to HAp nanoparticles respectively of HApWW nanoparticles (Figure 1a,c). The non-uniform distribution of the nanoparticles covered with EOs (Figure 1d) in comparison with the uncovered ones (Figure 1b) can be observed.

The 3D surface plots of the SEM images of HAp and HApWW analyzed samples (Figure 1b,d) seem to indicate a change in the porosity of HApWW sample. In our previous paper [25] it was showed by SEM investigations that the porosity of HAp nanoparticles was increased in the case of particles covered with lavender essential oil. It was explained by a good adsorption of lavender EO on the surface of HAp particles.

The surface topology of the HAp and HApWW samples was also investigated using AFM studies. In order to be investigated the HAp and HApWW powders were pressed into pellets. The results of the AFM studies are presented in Figure 1e–h. The surface of the HAp pellet was homogeneous with a roughness (R_RMS_) of 6.93 nm. The surface topography of the HApWW was also homogenous with a roughness above 10 nm having a R_RMS_ value of 13.53 nm. The AFM micrographs evidenced that the WW EO had an influence on the HAp nanoparticles morphology, emphasizing that the HApWW pellet’s surface topology is comprised of larger grains than the HAp’s pellet surface topology.

**Figure 2:**
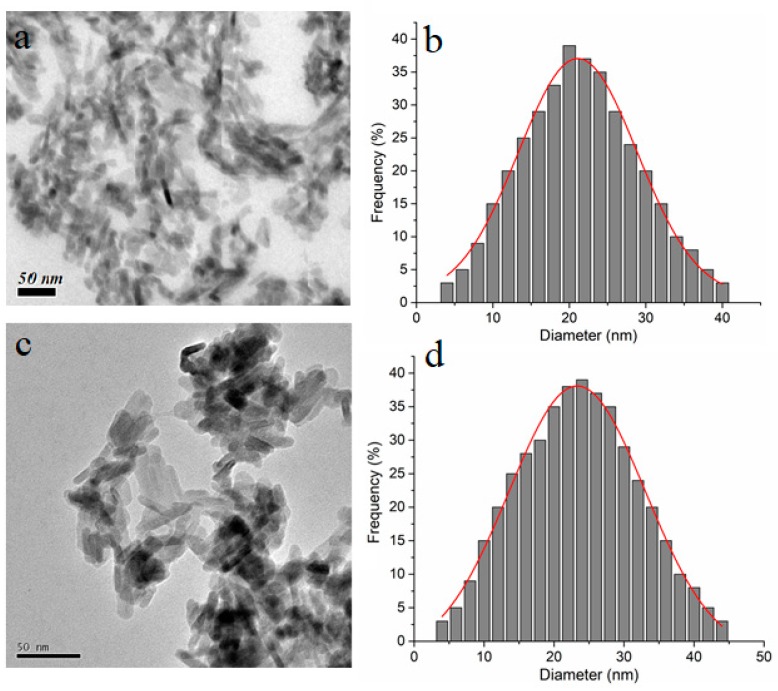
TEM image of HAp (**a**) and HApWW (**c**) nanoparticles. Distribution of HAp (**b**) and HApWW (**d**) nanoparticles obtained from TEM images.

TEM image of pure hydroxyapatite and hydroxyapatite coated with wormwood (*Arthemisia absinthium* L.) essential oil (HApWW) were obtained to observe the morphology of nanoparticles and to make particles size distribution as shown in Figure 2. The HAp nanoparticles observed in Figure 2a showed an ellipsoidal shape. HApWW nanoparticles showed a regular morphology (Figure 2c) preserving the ellipsoidal shape of HAp nanoparticles. The mean diameter calculated from the TEM images (D_TEM_) was 20.6± 1.5 nm for HAp nanoparticles (Figure 2b) while for HApWW nanoparticles the D_TEM_ was 23.31 ± 2.3 nm (Figure 2c).

**Figure 3:**
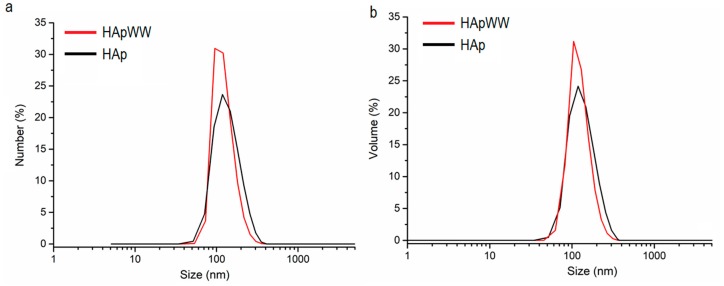
Dynamic light scattering (DLS) measurements of size dispersion of HAp (black) and HApWW (red) represent data weighted by number (**a**) and volume (**b**) of particles.

Particle size measured by DLS was established for the HAp and HApWW nanoparticles in suspension (Figure 3a–b). Figure 3 revealed DLS data weighted by numbers (Figure 3a) and volume (Figure 3b) of HAp and HApWW particles obtained. From the DLS data weighted by numbers the D_H_ was 120.4 ± 5.5 nm for HAp nanoparticles in suspensions and 103.5 ± 6.2 nm for HApWW nanoparticles in suspensions (Figure 3a). On the other hand, the D_H_ was 122.8 ± 4.2 nm for HAp nanoparticles in suspensions and 105.4 ± 5.8 nm for HApWW nanoparticles in suspensions from the DLS data weighted by volume (Figure 3b). However, HApWW nanoparticles present slightly narrower size distribution than the HAp sample. The number weighted DLS measurement of the samples and the volume-weighted exhibits one peak for both samples analyzed. In Figure 3 it can be seen that the obtained nanoparticles of HAp and HApWW have unimodal particle size distributions, both in number and in volume. The DLS size determined (D_H_) was larger than size evaluated by the TEM, as expected. The disagreement, between D_H_ and D_TEM_ is because DLS measures a hydrodynamic size, rather than otherwise a physical size [26]. On the other hand, the stability of HAp and HApWW nanoparticles is different. The large size observed by DLS could also be due to the agglomeration mode of the particles in suspension. Particles coated with essential oil can lead to better stability of the nanoparticles particles in suspension and a smaller agglomeration. On the other hand, it is quite possible that the difference between the diameters of the two HAp and HApWW samples obtained from DLS measurements is due to the presence of an adsorption layer, which is composed of essential oil.

**Figure 4:**
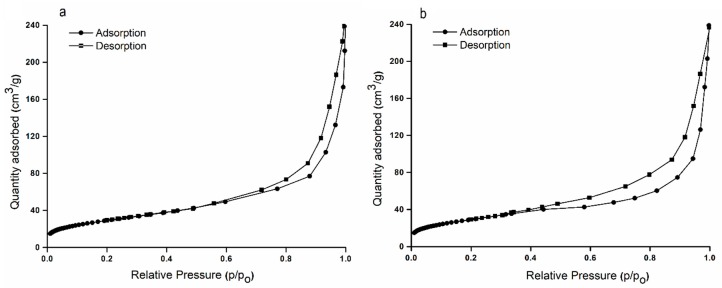
The nitrogen adsorption/desorption isotherms of HAp (**a**) and HApWW (**b**) samples.

The nitrogen adsorption/desorption isotherms of HAp and HApWW samples are exhibited in Figure 4a–b. It can be seen that the respective N_2_ adsorption/desorption isotherms of HAp and HApWW samples presented typical type IV isotherms in agreement with the International Union of Pure and Applied Chemistry (IUPAC) classification [27]. The obtained HAp and HApWW samples presented a distinct hysteresis loop, denoting that the samples have a typical behavior of mesoporous materials [27]. Following these studies important information was obtained regarding single point surface area, BET surface area, Langmuir surface area and t-plot external surface area of HAp and HApWW samples. These values are shown in Table 2. The results obtained by the nitrogen adsorption/desorption are in agreement with results of SEM, AFM and DLS investigations. Furthermore, the correlation of these results is also supported by previous studies by A. Wypych [28].

**Figure 5:**
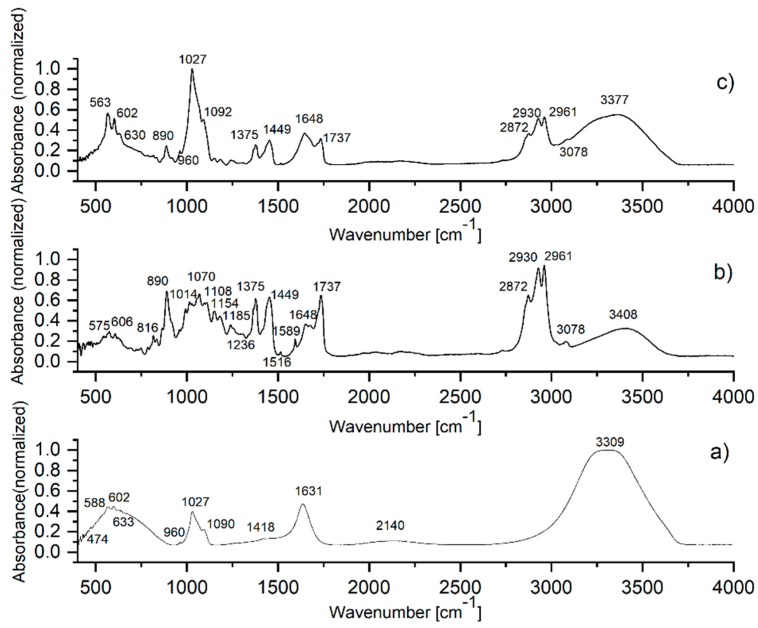
FTIR spectra of the HAp solution (**a**), WW EO (**b**) and HApWW (**c**) samples.

The IR spectra and absorption band wavenumbers of HAp, WW EO and HApWW samples are presented in Figure 5a–c and Table 3.

The molecular spectra were normalized from 0 to 1. The IR spectrum of the WW EO and its influence on the molecular structure of HAp are presented in Figure 5b,c, while main absorption molecular bands of the HAp are identified in Figure 5a. The assignments and wavenumber position of the HAp, WW EO and HApWW IR bands are shown in Table 3. The fundamental vibrational modes of the [PO_4_]^3−^ groups of the HAp sample are manifested at 474 cm^−1^ (ν_1_), 588, 602, 633 cm^−1^ (ν_4_), 960 cm^−1^ (ν_1_), 1027, 1090 cm^−1^ (ν_3_) [29,30]. The [CO_3_]^2−^ vibrational carbonate group presents a small IR peak at 1418 cm^−1^. The [CO_3_]^2−^ group absorption band appear in the HAp water solution IR spectrum as carbonated groups are formed during the chemical synthesis of the HAp nanoparticles. The IR bands from 1631 and 3309 cm^−1^ are attributed to the O–H vibrations in the water molecules absorbed into the HAp structure. The C-O stretching vibrations are identified at 1014, 1070, 1108, 1154, 1185, 1236 cm^−1^ while the peaks characteristics to C=O appear at 1737 cm^−1^. The C=C-C stretching vibrations are manifested at 1516 and 1589 cm^−1^. The C-H vibrational groups coupled in different chemical configurations present specific IR bands in 2800–3400 cm^-1^ and 700–1500 cm^−1^ spectral ranges (see Table 3) [31,32,33,34,35].

The antioxidant and antibacterial properties of *A. absinthium* L. essential oil and extracts and the antimicrobial mechanisms involved, were reported in several papers. In his work, Wright [36] reported that the wormwood stimulant property is due to the existence of bitter substances such as artabsin (tricyclic sesquiterpene lactone) and absinthin (dimer of sesquiterpene lactone) which are present in the plant extracts. In their study, Msaada et al. [9], reported that the essential oils of wormwood obtained from plants cultivated in different regions showed an interesting antimicrobial activity and concluded that the antimicrobial activity of the essential oil is dependent on the organoleptic quality of the oil, which is strongly correlated to the collection region of the plants. In this context, the antimicrobial properties of wormwood essential oil extracted from the root parts of the *A. Absinthium* L. plants and the antimicrobial properties of hydroxyapatite coated with the wormwood essential oil were studied against a collection of Gram-positive (*S. aureus* ATCC 25923, *S. aureus* ATCC 6538, *S. aureus* 388 (MRSA), *E. faecium* DSM 13590) and Gram-negative bacteria (*E. coli* ATCC 25922, *E. coli* C5, *P. aeruginosa* ATCC 27853, *P. aeruginosa* ATCC 9027) and yeast (*C. parapsilosis* ATCC 22019).

The evaluation of the antimicrobial activity was performed using two adapted standard approaches, MIC and MBC methods, using 96 well plates with two-fold dilutions of the WW EO and HApWW samples. The values for the minimum inhibitory concentration (MIC) and minimum bactericidal concentration (MBC) obtained for DMSO, HAp, WW EO and HApWW are presented in Table 4. The MIC values obtained in the case of WW EO were in the intervals of 7.81 µL/mL to 62.5 µL/mL. The most sensitive to the WW EO was proven to be the *C. parapsilosis* fungal strain with the MIC and MBC values of 7.81 µL/mL. The lowest MIC and MBC values for the HApWW were also obtained for the *C. parapsilosis* fungal strain (15.62 µL/mL). The results emphasized that the WW EO was the most efficient against the *C. parapsilosis* fungal strain. Furthermore, the results obtained for the MIC and MBC values in the case of WW EO and HApWW, HAp and DMSO indicated that the WW EO had a strong antimicrobial effect against the tested microbial strains and also that the antimicrobial properties of HApWW are due to the presence of WW EO. Moreover, the results of MBC studies emphasized that the tendency of the antimicrobial properties of the tested samples were the following *C. parapsilosis* ATCC 22019 > *E. faecium* DSM 13590 > *E. coli* ATCC 25922 > *P. aeruginosa* ATCC 27853 > *P. aeruginosa* ATCC 9027 > *E. coli* C5 > *S. aureus* ATCC 25923 > *S. aureus* ATCC 6538 > *S. aureus* 388 (MRSA).

The results highlighted that the use of DMSO as solvent did not affect bacterial growth. The results regarding the antimicrobial activity of HAp solution and DMSO are in agreement with previously reported studies by the authors [20,25]. Even though, the pharmacologic effects of DMSO are diverse and it has been reported to present anti-inflammatory, cryopreservative, antiischemic, radioprotective qualities and even antiseptic properties [37] its effect on microorganisms depends on numerous factors such as microbial strain, concentration, pH, etc [38]. In this context, when used as solvent in antimicrobial assays its effects against the tested strains is also of great importance [38]. The effect of the DMSO/water solution against various microorganisms such as *Candida albicans*, *Aspergillus niger*, *Escherichia coli*, *Staphylococcus epidermidis*, *Staphylococcus aureus* MRSA (BMB9393), etc. was evaluated by Lopes-Lutz et al. [39] in their study. The results presented by Lopes-Lutz et. al. revealed an absence of inhibition zones which confirmed the non-toxic effect of the DMSO/water solution against the tested microorganisms. The lack of antimicrobial properties of DMSO was also reported in other studies conducted by Janaki et al. [40]. Moreover, the data suggested that HAp did not presented any inhibitory effect against the tested microbial strains, except for *C. parapsilosis* ATCC 22019. These results could be attributed to the fact that fungal cells have ergosterol in their membrane that enables them to make various gradients between cytoplasmic membranes keeping their membrane uniformity. Ergosterol is the main sterol component of fungal cell membranes and it plays a crucial role for the normal growth of cells. It has the function of regulating membrane fluidity, asymmetry and membrane integrity. It is also involved in the proper function of membrane-bound enzymes, making it vital in cell survival. Among other, he is responsible for the H+-ATPase content that maintains the electrochemical proton gradient across the cell membranes necessary for nutrient uptake [41]. Therefore, the use of any NPs could lead to the destabilization of these gradients and to the membrane conformity, leading thus to the destruction of the membrane and to cause cell death. Moreover, the most pronounced antifungal effect was obtained in the case of the WW EO demonstrating that even though, the fungal strain is susceptible to HAp, the antifungal properties of WW EO are stronger. Moreover, it was emphasized that the synergistic mechanisms occurring between the components of the essential oils have an important role in determining their antimicrobial effects. Usually in the conventional antimicrobial therapies, there are known and studied three bacterial targets: the cell wall synthesis, translational machinery, and DNA replication machinery [40]. During the years, it has been reported that the microorganism has the means of developing resistance against each of these modes of action. Nonetheless, these resistance mechanisms are irrelevant when nanoparticles (NPs) are involved due to the fact that their mode of action involves a direct contact with the bacterial cell wall, without the need to penetrate the cell. The major processes responsible for the antimicrobial effects of NPs were described as being the disruption of the bacterial cell membrane, the generation of reactive oxygen species (ROS), the penetration of the bacterial cell membrane and the induction of intracellular antibacterial effects. An important role is played by the bacterial cell. Studies have shown that nanoparticles in general have a significantly increased antibacterial effect against Gram-positive bacteria than against Gram-negative bacteria, because the cell wall of Gram-negative bacteria is composed of LPS, lipoproteins, and phospholipids, that form a barrier permeable only to macromolecules. Nonetheless, the mechanisms involved in the bacterial death is dependent on the components and structure of the bacterial cell [42,43,44,45]. Our results suggested that the samples were most efficient against *C. parapsilosis* ATCC 22019, *E. faecium* DSM 13590, and *E. coli* ATCC 25922, being in agreement with the literature data [42,43,44,45]. Furthermore, the results of the antimicrobial activity against the *S. aureus* tested bacterial strains obtained depicted that the antimicrobial properties of the HApWW and WW EO had the following tendency *S. aureus* ATCC 25923 > *S. aureus* ATCC 6538 > *S. aureus* 388 (MRSA). *S. aureus* is one of the most common bacterial agent that causes invasive infections. The resistance of methicillin-resistant *Staphylococcus aureus* strains is usually attributed to a nonnative gene that could encode a penicillin-binding protein (PBP2a) which allows cell-wall biosynthesis, to be achieved even in the presence of conventional antibiotics [46]. Moreover, other bacterial strains that are usually encountered in hospital related infections such as *E. coli* had developed resistance to conventional drugs. In the case of *E. coli* (carbapenemase producer strain), the resistance is attributed to the cells ability to produce an enzyme called a carbapenemase that disables the drug molecule [47]. Even though there have been numerous studies trying to elucidate the mechanisms of microbial cells resistance to antimicrobial drugs, there are still not fully understood and there should be further investigations. In this context, the results of the antimicrobial assays obtained for the tested microbial strains revealed that the reference standard microbial strains obtained from the ATCC were more susceptible to the HApWW and WWEO compounds than their resistant counterparts were. Therefore, the results suggested that *E. coli* ATCC 25922 was strongly inhibited by both HApWW and WWEO compared to *E. coli* C5 strain. The same behavior was also observed in the case of *S. aureus* bacterial strains. The two ATCC *S. aureus* strains tested were more susceptible to the tested compounds then the *S. aureus* 388 (MRSA) strain. In this case, the tested samples had a greater inhibitory effect on the reference strain *S. aureus* ATCC 25923 than in the case of the methicillin-sensitive *S. aureus* ATCC 6538 strain. Similar results were obtained in the case of the two *P. aeruginosa* tested strains. *P. aeruginosa* ATCC 27853 and *P. aeruginosa* ATCC 9027 bacterial strains, used in this study, have the ability to degrade aliphatic hydrocarbons but can be characterized as having either fast (ATCC 27853) or slow (ATCC 9027) rates of growth. This difference between these two strains could be the reason for the different susceptibility of the bacterial cells towards the same antimicrobial agents [48]. Moreover, the results have emphasized that the most pronounce antimicrobial activity was obtained for the WW EO samples, making it obvious that the essential oil through its chemical constituents have significant influence against any type of microorganism, from fungal cells to Gram positive and Gram-negative bacterial cells.

The response of the microbial membrane of the tested microorganism when exposed to WW EO and HApWW was also evaluated using a flow cytometric approach. DiBAC4(3) is a translational membrane potential dye that has the ability to redistribute within the bacterial cell membrane when the membrane potential suffers changes. The intensity of the dye’s fluorescence is enhanced when the membrane depolarization happens. The flow cytometry studies performed in association with the DiBAC4(3) green fluorescent dye allowed the determination of the percentage of depolarized microbial cells. The results are presented in Table 5. The results revealed an increase of the fraction of depolarized microbial cells in the case of *E. coli* ATCC 25922, *P. aeruginosa* ATCC 27853 and *S. aureus* ATCC 25923, *E. faecium* DSM 13590 and *C. parapsilosis* ATCC 22019 when were exposed to WW EO at MIC concentrations ranging from 2.1% for *E. faecium* DSM 13590 to 34.2% for *P. aeruginosa* ATCC 27853 comparative with the control microbial cells, for which the increase in the fraction of depolarized microbial cells ranged from 0.0% for *E. coli* ATCC 25922 to 1.3% for *C. parapsilosis* ATCC 22019. Furthermore, an increase of the fraction of depolarized microbial cells was also noticed in the case of the microbial cells treated with HApWW, ranging from 0.2% for *E. coli* ATCC 25922 to 14.1% for *C. parapsilosis* ATCC 22019. On the other hand, a considerably increase in the green fluorescence of *P. aeruginosa* ATCC 27853 microbial cells versus the control was observed only in the case of treatment with WW EO.

The results regarding the antimicrobial activity of WW EO and HApWW against a collection of Gram-positive (*S. aureus* ATCC 25923, *S. aureus* ATCC 6538, *S. aureus* 388 (MRSA), *E. faecium* DSM 13590) and Gram-negative bacteria (*E. coli* ATCC 25922, *E. coli* C5, *P. aeruginosa* ATCC 27853, *P. aeruginosa* ATCC 9027) and yeast (*C. parapsilosis* ATCC 22019) obtained in this study have emphasized that both WW EO and HApWW possess good antimicrobial activity and that they can be furthered considered in the development of new antimicrobial agents. The results are in good agreement with other existing studies. Even though, there are numerous studies on the antimicrobial activity of EOs, results on the existing synergies between the chemical components of essentials oils are still limited. It was reported that the antimicrobial activity of an EO could be attributed not only to its separate constituents but also to the synergies that appear between the major and minor constituents present in the composition of the EO [49]. Therefore, the antimicrobial properties obtained in our studies could be attributed both to the interactions between the interactions of the WW EO constituents, as well as to the synergy that formed between the EO’s chemical components with the hydroxyapatite structure. The oxygen-containing functional groups of WW EOs could generate effective bonding between WW EOs and HAp. The Ca^2+^ could be captured by O-C=O groups and could lead to chemical bonding between O-C=O groups of WW EOs and the Ca^2+^ in HAp. On the other hand, according L. Zhang et al. [50], the Ca^2+^ could also be attracted by C=O and C-O groups through electrostatic force. Moreover, during the HApWW synthesis the Ca^2+^ it could be attracted by carboxyl groups using the following reaction:

-COO^−^ + Ca^2+^ + ^-^OOC → -COO^−^ · · · Ca^2+^ · · · -COO^−^ (1)

Furthermore, various studies have evidenced that also biomaterial surface roughness and particle morphology of the materials are relevant properties when dealing with the bacterial adhesion process [51,52]. It was highlighted that the existence of surface irregularities appeared due to the surface roughness could promote bacterial adhesion and consequently biofilm formation by inducing their attachment [53]. Nonetheless, even though there have been several studies reporting that there is indeed a positive correlation between bacterial adhesion and an increased surface roughness [54,55], some researchers showed that there is no correlation between the ability of bacterial cells to adhere to a surface and the irregularities or roughness of the surface [56]. Moreover, this matter is still being disputed and there are reports with different results for various particular roughness parameter, bacterial specie and physico-chemical properties of the samples. Ours results suggested that the HApWW samples exhibited antimicrobial properties and HAp samples did not, with the exception of *C. parapsilosis* ATCC 22019 strain, even though according to AFM studies, their surface roughness was higher than the one obtained for HAp samples. Furthermore, the HApWW samples also exhibited a higher BET surface area compared with HAp samples, and higher particle size according to TEM results. These results are in agreement with previously reported studies [55,56,57] and clearly suggest that the antimicrobial activity of the HApWW samples are a consequence of multiple factors such as their sizes, surface area, roughness, active antimicrobial substances and also the cell morphology of the tested microorganisms. The results obtained from the GC-MS analysis revealed that WW EO is mainly constituted of monoterpenes. These are a class of terpenes consisting of two isoprene units and having the molecular formula C_10_H_16_. Considering their properties and their chemical structure, one of the proposed mechanism in the forming of HApWW samples would be through hydrogen bonding and/or dipole interactions. Furthermore, the antimicrobial activity presented by the WW and HApWW samples is strongly correlated with the presence of the major constituents α-Fenchene, Sabinene, β-Thujene, β-myrcene and methyl salicylate, which were reported to possess good antimicrobial activity against microbial strains such as *E. coli, B. subtillis, P. aeruginosa, S. cerevisiae* and *C. albicans* [57]. Moreover, the studies reported by Ghaffari et al. [58] regarding EOs having most abundant chemical constituents β-pinene, β-myrcene, limonene, and caryophyllene also demonstrated antimicrobial activity against some highly susceptible strains such as *E. coli*, *C. albicans*, and *S. aureus.* Furthermore, the antimicrobial activity of the investigated samples could also be attributed to the less abundant constituents of the WW EO such as 1,8-Cineole, γ-Terpinene and β-Pinene which were previously reported as having antimicrobial activity against a wide range of microbial strains [59,60]. These studies confirm our results and emphasize that the antimicrobial activity of the investigated samples is strongly correlated not only with the chemical constituents but it is also influenced by the synergies developed between the chemical constituents of the EO between themselves and also with HAp structure. Moreover, Msaada et al. [9], reported that wormwood EOs had a good inhibitory activity against three phytopathogenic microorganism (*F. graminearum, F. culmorum, and F. oxysporum).* In their study, Msaada et al. [9] demonstrated that essential oils of wormwood obtained from plants from different region had a different antimicrobial behavior and that the highest inhibitory activity was obtained against *Staphylococcus aureus* bacterial strain. Furthermore, Kordali et al. reported that wormwood EOs extracted from plants originally from Turkey, which had as main components camphor, 1,8-cineole, and chamazulene exhibited fungicidal effects against 34 species of fungi [61]. Additionally, Umpierrez et al. [62] showed in their study that the EO obtained from *A. absinthium* plants from Uruguay, which were rich in thujone, exhibited antifungal activity against *Alternaria sp*. and *Botrytis cinerea*.

Nonetheless, supplementary research is needed to be able to identify and confirm the compounds present in wormwood essential oil, which are responsible for their antimicrobial effects.

## 3. Materials and Methods

### 3.1. Sample Preparation

The essential oil (EO) used in this study was wormwood (*Arthemisia absinthium* L.) essential oil (WW EO). The essential oils were extracted from the root parts of *A. absinthium*, collected in the preflowering, stage from plants grown in a local farm situated in the southeast of Romania (20 May 2016). After being harvested, the root parts of the plants were dried at room temperature, packed in paper bags, and then stored in a dry place until oil extraction. The essential oil of wormwood was obtained by steam distillation method. For this purpose, 200 g of dried plant was used for the extraction of 0.2 mL EO.

Hydroxyapatite, (HAp) solutions were synthesized using an adapted coprecipitation method. Throughout the synthesis the molar ratio Ca:P was kept at 1:67 [63]. The precursors used in the HAp synthesis were calcium nitrate (Ca(NO_3_)_2_∙4H_2_O, Sigma Aldrich, St. Louis, MO, USA), ammonium hydrogen phosphate ((NH_4_)_2_HPO_4_; Wako Pure Chemical Industries Ltd., Richmond, VA, USA), ammonium hydroxide (NH_4_OH, Wako Pure Chemical Industries Ltd., Richmond, VA, USA), and double-distilled water. (NH_4_)_2_HPO_4_ and Ca(NO_3_)_2_·4H_2_O were dissolved in ethanol and mixed for 2 h at 40 °C. To the solution of obtained calcium nitrate solution were added 2 mL of essential oil. To the solution containing calcium nitrate and essential oil, the solution containing ammonium hydrogen phosphate was added drop by drop. The resulting solution was stirred for 12 h at 40 °C. The NH_3_ was added during synthesis to maintain the pH at a value of 10. The resulting solution was centrifuged and the resulting precipitate was redissolved in ethanol. After the last centrifugation, the resulting precipitate was redispersed into an essential oil solution in ethanol and stirred for 24 h at room temperature. The final solution obtained was noted HApWW.

### 3.2. Physico-Chemical Characterization

The chemical constituents of the wormwood essential oil extracted from root parts of *Arthemisia absinthium* L. samples were determined by gas chromatography (GC) analysis using a Perkin Elmer gas chromatographer (Perkin Elmer Inc, Waltham, MA, USA). The gas chromatographer was equipped with a flame ionization detector (FID) (PerkinElmer Inc, Waltham, MA, USA). The measurements were conducted as previously described in Predoi et al. [35]. Additionally, a Perkin-Elmer Turbomass Quadrupole mass spectrometer (PerkinElmer Inc., Waltham, MA, USA) was used for the GC/MS studies [20]. The chemical elements of the studies sample were identified using NIST and Wiley Registry 8 Edition mass database, n-alkalene (C_9_-C_22_) hydrocarbon series (Nile, Italy). Mass spectra found in the literature [64,65] were also used for the identification of the chemical components. The results were presented as relative area percent (%).

The surface morphology features of the *Artemisia absinthium* essential oil, HAp nanoparticles and *Artemisia absinthium-HAp nanoparticles*, were investigated by scanning electron microscopy (SEM) using a HITACHI S4500 microscope. The HAp nanoparticles and *Artemisia absinthium-HAp nanoparticles* were prepared on a conductive carbon tape with double adhesion and introduced in the microscopy. A drop of *Artemisia absinthium* essential oil was dried and then analyzed. Using a 5 kV electron acceleration voltage and an Everhart-Thornley detector (ETD) the samples were analyzed and their characteristic SEM images were acquired. Moreover, the surface morphology of the powders was also analyzed using atomic force microscopy (AFM), in non-contact mode, with the help of an NT-MDT NTEGRA Probe NanoLaboratory instrument (NT-MDT, Moscow, Russia). For this purpose, the powders were pressed into pellets and their surface morphology was investigated with the aid of a silicon NT-MDT NSG01 cantilever coated with a 35 nm gold layer. The AFM micrographs were acquired on surface areas of 2.5 × 2.5 µm^2^, and the root mean square roughness (R_RMS_) was also presented.

The morphology of the samples was also investigated using transmission electron microscopy with a CM 20 (Philips-FEI, Hillsboro, OR, USA) transmission electron microscope with a Lab6 filament (Agar Scientific Ltd., Stansted, UK) operating at 200 kV.

The hydrodynamic diameter of the samples was investigated by Dynamic Light Scattering (DLS) using dynamic light scattering (SZ-100 Nanoparticle Analyzer, HORIBA, Ltd., Kyoto, Japan) at 25 ± 1 °C. All the samples were diluted in distilled water before analysis.

The textural characteristics of the obtained samples were investigated by low temperature N_2_ adsorption desorption using a Micromeritics ASAP 2020 Physisorption Analyzer (Micromeritics Instrument Corp., Norcross, GA, USA).

FTIR analysis of HAp, *Artemisia absinthium* essential oils and HAp essential oil samples was performed using a Perkin Elmer spectrometer (Waltham, MS, USA) SP-100 with a 4 cm^−1^ resolution, in the 400–4000 cm^−1^ spectral range. The transmission IR spectra were acquired with an Attenuated Total Reflection (ATR) accessory. Using the A = log (1/T) formula, the transmission spectra were converted into absorption spectra using the SPECTRUM software (Version 6.4.1Perkin Elmer, Waltham, MS, USA) [25,66].

### 3.3. Antimicrobial Assays

#### 3.3.1. Microbial Strains and Culture

The antimicrobial assays of the HAp, WW EO and HApWW samples were conducted using both reference and clinical microbial strains from the Department of Microbiology, Faculty of Biology, University of Bucharest. The microbial strains used in this study were Gram-negative bacteria (*E. coli* ATCC 25922, *E. coli* C5 (carbapenemase producer strain), *P. aeruginosa* ATCC 27853, *P. aeruginosa* ATCC 9027), Gram-positive bacteria (*S. aureus* ATCC 25923, *S. aureus* ATCC 6538, *E. faecium* DSM 13590), and the fungal strain *C. parapsilosis* ATCC 22019.

#### 3.3.2. Agar Diffusion Method

The qualitative assay regarding the antimicrobial activity of the WW EO and HApWW was performed using an adapted agar diffusion method [67,68,69,70]. 0.5 McFarland inoculum of the tested microbial strains were prepared from 18–24 h cultures and inoculated in Muller Hinton agar plates. Afterwards, a volume of 10 µL of the tested samples was deposited on the agar surface and the Petr dishes were incubated in aerobic conditions at 37 °C for 24 h in order to determine the diameters of the inhibition growth zones.

#### 3.3.3. Broth Micro-Dilution Assay

The antimicrobial properties of the WW EO and HAp coated with WW EO was conducted by broth micro-dilution assay. For this purpose, the WW EO, HApWW and HAp samples were solubilized in dimethyl sulfoxide (DMSO) (ratio (*v*/*v*) 1:1) and the solution was used to obtain serial two-fold dilutions in MHB (Muller Hinton Broth), in 96 well plates. Microbial suspension of 10^6^ CFU mL^−1^ were obtained from 24 h solid cultures and used for inoculation of each well containing two-fold dilutions WW EO, HApWW and Hap and also for the inoculation of the growth control wells (positive control). The antimicrobial activity of the solvent (DMSO) was also evaluated. All assays were done in triplicate.

The minimum inhibitory concentration (MIC) values and minimum bactericidal concentration (MBC) were determined using conventional plating method after 18–24 h incubation of the inoculated microtiter. The MIC value was considered the lowest concentration for which visible inhibition of the bacterial growth was observed after overnight incubation at 37 °C. In addition, the MBC value was considered the lowest concentration required to stop bacterial growth in the MHA plates after overnight incubation at 37 °C.

#### 3.3.4. Flow Cytometric Testing

The influence of the WW EO, HApWW and HAp samples on the microbial membrane was also evaluated with flow cytometry using a potential-sensitive fluorescent dye DiBAC4(3). For this purpose, the microbial cells at obtained MIC concentrations were cultivated in MHB in the presence the tested samples. After 24 h incubation at 37 °C in aerobic conditions, the microbial cultures were stained with a 0.5 μg/mL DiBAC4(3) (Invitrogen/Life technologies, Carlsbad, USA) solution. After incubation in the dark at room temperature, the intensity of the fluorescence (IF) was determined using an Accuri C6 plus flow cytometer (Becton Dickinson, Biosciences). Non-treated and non-stained cells were used for each microbial strain in order to evaluate the native cell auto-fluorescence and to define the acquisition settings. Data acquisition and analysis were carried out using the BD Accuri C6 plus software.

## 4. Conclusions

This research aimed to achieve an *Artemisia absinthium* essential oil-hydroxyapatite composites and to highlight its antibacterial activity. The presence of the essential oil in the biocomposites based on the essential oil and hydroxyapatite was evidenced by FTIR studies which showed that the molecular bands of *Artemisia absinthium* EO are superimposed with the IR bands characteristic of the HAp structure. Following this study, we have shown that wormwood essential oil has antibacterial activity against the tested strains *C. parapsilosis* ATCC 22019 > *E. faecium* DSM 13590 > E. coli ATCC 25922 > *P. aeruginosa* ATCC 27853 > *P. aeruginosa* ATCC 9027 > *E. coli* C5, *S. aureus* ATCC 25923 > *S. aureus* ATCC 6538 > *S. aureus* 388 (MRSA). The antimicrobial activity of biocomposites based on hydroxyapatite and essential oil is due to the presence of the essential oil. HAp showed no inhibitory effect against the tested microbial strains, except for *C. parapsylosis* ATCC 22019. The results of this study show that both wormwood essential oil and biocomposite based on hydroxyapatite and essential oil could be used to treat microbial infections. Due to their biocompatibility combined with antimicrobial properties these types of composites based on hydroxyapatite and essential oils could be suitable for the development of new applications in cosmetics, pharmaceutical, medical or food industry. In addition, they could play an important role in the biomedical field for developing wound dressings, antimicrobial sprays as well as ointments or gels with antimicrobial properties.

## Figures and Tables

**Table 1:** Chemical composition of wormwood essential oils (WW EOs) obtained from dried root parts of *Arthemisia absinthium* L.

Compounds	Percentage of Chemical Composition (%)
α-Fenchene	52.29
Sabinene	7.2
β-Thujene	6.28
β-myrcene	5.47
methyl salicylate	4.2
endo-bornylacetate	2.21
ϒ-isogeraniol	1.95
neryl isobutyrate	1.9
dyhidrocarveol acetate	1.29
nerol	1.28
1,8-Cineole	1.05
ϒ-Terpinene	1.05
β-Pinene	1.02
camphene	tr
α-pinene	tr
hexanol	tr
limonene	tr
neryl acetate	tr
bornyl isovalerate	tr
neryl propionate	tr

tr*-trace amounts.

**Table 2:** Parameters of nitrogen adsorption/desorption isotherms of HAp and HApWW samples.

Sample	Single Point Surface Area (nm)	BET Surface Area (nm)	Langmuir Surface Area (nm)	t-Plot External Surface Area (nm)
HAp	101.12	102.54	153.50	112.10
HApWW	115.24	116.72	173.40	123.60

**Table 3:** Assignment of IR bands identified in the IR spectra of HAp, WW EO and HApWW.

HAp Solution IR Band Wavenumber	IR Band Assignment	WW EO IR Band Wavenumber	IR Band Assignment	HApWW IR Band Wavenumber	IR Band Assignment
588, 602, 633	vibrations in [PO_4_]^3−^	816, 890	C-H vibrations	563, 602, 630	vibrations in [PO_4_]^3−^
1418	vibrations in [CO_3_]^2−^	1014, 1070, 1108, 1154, 1185,1236	C-O vibrations	890	C-H vibrations
960, 1027, 1090	vibrations in [PO_4_]^3−^	1375,1449	C-H vibrations	960, 1027, 1092	vibrations in [PO_4_]^3−^
1631, 3309	O-H vibrations	1516,1589	C=C-C stretching vibrations	1375, 1449	C-H vibrations in CH_3_
2140		1648	C=C vibrations	1648	C=C vibrations
		1737	C=O stretching vibrations	1737	C=O stretching vibrations
		2872, 2961	C-H vibrations in CH_3_	2872, 2961	C-H vibrations in CH_3_
		2930	C-H vibrations in CH_2_	2930	C-H vibrations in CH_2_
		3078	C-H vibrations	3078	C-H vibrations
		3408	O-H vibrations	3377	O-H vibrations

**Table 4:** MIC and MBC (µl/mL) values for WW EO HApWW, HAp and DMSO.

Tested EOs	WW EO	HApWW	HAp	DMSO
**Bacterial Strains**				
*E. coli* ATCC 25922	MBC 125 MIC 31.25	125 125	> 50	>250
*E. coli* C5	MBC 125 MIC 62.5	125 62.5	>250	>250
*P. aeruginosa* ATCC 27853	MBC 62.5 MIC 62.5	62.5 62.5	>250	>250
*P. aeruginosa* ATCC 9027	MBC 62.5 MIC 62.5	62.5 62.5	>250	>250
*S. aureus* ATCC 25923	MBC 62.5 MIC 62.5	250 62.5	>250	>250
*S. aureus* ATCC 6538	MBC 62.5 MIC 62.5	250 62.5	>250	>250
*S. aureus* 388 (MRSA)	MBC 62.5 MIC 62.5	250 62.5	>250	>250
*E. faecium* DSM 13590	MBC 250 MIC 31.2	250 31.2	>250	>250
*C. parapsilosis* ATCC 22019	MBC 7.81 MIC 7.81	15.62 15.62	250 125	>250

**Table 5:** The percentage of depolarized bacterial cells treated with WWEO and HApWW at MIC values.

Samples	WW EO	HapWW	Control
Bacterial Strains			
*E. coli* ATCC 25922	9.7%	0.2%	0%
*P. aeruginosa* ATCC 27853	34.2%	5.4%	0.1%
*S. aureus* ATCC 25923	3.5%	0.9%	0.6%
*E. faecium* DSM 13590	2.1%	8.2%	0.3%
*C. parapsilosis* ATCC 22019	6.4%	14.1%	1.3%
